# Performance of the EQ-5D and the EQ-5D+C in elderly patients with cognitive impairments

**DOI:** 10.1186/1477-7525-5-33

**Published:** 2007-06-14

**Authors:** Claire AG Wolfs, Carmen D Dirksen, Alfons Kessels, Daniëlle CM Willems, Frans RJ Verhey, Johan L Severens

**Affiliations:** 1 Department of Clinical Epidemiology and Medical Technology Assessment, Maastricht University Hospital, Maastricht, Netherlands; 2 Department of Psychiatry & Neuropsychology, Maastricht University, Maastricht, Netherlands; 3 Department of Health Organisation, Policy and Economics, Maastricht University, Netherlands

## Abstract

**Background:**

The EQ-5D is a reliable tool for measuring Health-Related Quality of Life (HRQoL). However, concern has been expressed that it may ignore elements of HRQoL, particularly cognition. In response to this concern, the EQ-5D has been extended with a cognitive dimension (EQ-5D+C). The aim of this study was to compare the performance of the EQ-5D and the EQ-5D+C in elderly patients with cognitive impairments by assessing their construct validity and responsiveness.

**Methods:**

Data from the MEDICIE study (n = 196) were used, in which all questionnaires were rated by proxies.

**Results:**

Regarding construct validity, we found similar correlations between the EQ-5D and the Mini Mental State Examination (MMSE) and between the EQ-5D+C and the MMSE. Furthermore, both the EQ-5D and the EQ-5D+C were responsive to changes in the MMSE, with the EQ-5D performing slightly better.

**Conclusion:**

We conclude that the EQ-5D performs well for evaluating HRQoL in a population with cognitive impairments. Based on the results of this explorative study, it does not seem necessary to adjust the current classification system by adding a cognitive dimension. However, in order to compare both instruments regarding utility values, it is necessary to develop a new scoring algorithm for the EQ-5D+C by conducting a general population study. Considering the explorative nature of this study, it is recommended that more aspects of the validity of both the EQ-5D and the EQ-5D+C are explored in patients with cognitive impairments using a more tailored study design.

## Background

The increasing number of older adults who are diagnosed with dementia has far-reaching implications for health service delivery and expenditures [1]. Economic evaluations are performed more often to assist decision-makers in setting priorities, especially with regard to resource allocation [2]. A central component of economic evaluations in health care is the use of preference-based instruments (also called value-based instruments) to measure changes in Health-Related Quality of Life (HRQoL). Preference-based measures, such as the EQ-5D [3,4], the SF-6D [5] and the HUI [6], are standardized multi-dimensional health state classifications [7]. For each of these instruments, health states have been valued using techniques such as standard gamble (SG) or time trade-off (TTO) [8]. These valuations were used for each instrument to generate a scoring algorithm of which a single utility score for each health state can be deduced.

The EQ-5D is commonly used to measure HRQoL and has been shown to be responsive, internally consistent and reliable in the normal population and other patient groups [9,10] as well as in patients with dementia [11,12]. However, concern has been raised that it may ignore elements of HRQoL of specific relevance to the elderly such as vision and hearing [1] and in particular cognition [1,13-17]. It is known that cognitive problems have an impact on personality, mood, behavior and global functioning [18], which are domains covered by the EQ-5D, but cognition might also be regarded as a separate dimension.

In response to the concern that the EQ-5D ignores cognition, the EQ-5D has been extended with a cognitive dimension (EQ-5D+C)[15]. In this study of Krabbe et al. (which was an adapted Dutch replication of the Global Burden of Disease study commissioned by the World Bank) [19], valuations (by means of a rating scale) elicited from EQ-5D+C descriptions were compared empirically with parallel EQ-5D descriptions in Dutch faculty members (i.e. scientific staff members and management members of the Department of Public Health, the Department of Clinical Epidemiology and Biostatics and the Institute of Social Medicine).

The EQ-5D+C generated different values compared with the EQ-5D. Whereas the content validity of the EQ-5D improved by adding cognition, both versions evoked equally reliable values. Based on these results, the authors emphasized the importance of considering the inclusion of a cognitive dimension. Furthermore, the EQ-5D+C was used to describe the health status of the Dutch population and to investigate sociodemographic differences [14].

In this study, the content validity also improved through the addition of the cognitive dimension, while the reliability remained unaltered. It was concluded by the authors that the EQ-5D+C is an efficient tool for establishing the health status in the community. Another way to examine if the EQ-5D should contain a cognitive dimension is to investigate the performance of the EQ-5D and the EQ-5D+C in a population with cognitive impairments. The aim of this explorative study was to compare the performance of the EQ-5D and the EQ-5D+C by assessing their construct validity and responsiveness in patients aged 55 and older with cognitive impairments.

## Methods

### Study population and data collection

Data were derived from the MEDICIE (Maastricht Evaluation of a Diagnostic Intervention for Cognitively Impaired Elderly) study. The MEDICIE study is a randomized controlled trial (RCT) comparing the effects of a multidisciplinary diagnostic observation center for psychogeriatric patients (DOC-PG) with care as usual on HRQoL, mental and physical health, and the costs and use of health care facilities by patients with psychogeriatric problems [20]. The DOC-PG is an outpatient facility, providing multidisciplinary assessment by somatic screening, psychogeriatric assessment, and evaluation of the required levels of care for the patient and his (her) carer. The main aim of the DOC-PG is to improve or maintain the HRQoL of patients.

In the MEDICIE study, a total of 234 patients and their caregivers agreed to participate and were included between July 2002 and October 2004. Randomization occurred at the level of general practices. The experimental group visited the new diagnostic facility (DOC-PG), whereas the control group was treated as usual, i.e. the GP made the diagnosis or referred the patient to a specialist facility, namely the Maastricht Memory Clinic (MMC) or the Department of Old Age Psychiatry of the Community Mental Health Service (RIAGG). Patients were followed up after 6 months and 12 months.

All outcome measures, except the Mini Mental State Examination (MMSE), were collected through personal interviews with the patient's proxy. After initial assessment by the aforementioned health care professionals, the baseline MMSE scores were gathered from the patient records. The researchers (C.W. and D.W) were trained to assess patients using the MMSE at the 6 and 12 month follow-up. When possible, follow-up scores by the professionals were used. Sociodemographic data of the patients (gender, age, living arrangements) and proxies (gender, age, relationship to patient) were collected at baseline. Diagnosis was established by the multidisciplinary teams working at the DOC-PG or the MMC/RIAGG respectively, and was based on the DSM-IV criteria or other regular criteria [21]. In this study, the baseline data and the data at the six and 12 month follow-up for the entire group were used since, for the purpose of this paper, it was not necessary to analyze the data of the control group and the experimental group separately.

### Instruments

#### Mini Mental State Examination (MMSE)

The MMSE is used to detect cognitive impairment, to assess its severity and to monitor cognitive changes over time [22]. The MMSE has a maximum score of 30 points, with different domains being assessed: orientation in regard to time and place (10 points), registration of three words (3 points), attention and calculation (5 points), recall of three words (3 points), language (8 points), and visual construction (1 point). Scores below 24 are considered abnormal and this is the cut-off used for dementia. Scores in the MMSE are often classified into different categories: 26–30 (normal ageing), 21–25 (mild dementia), 15–20 (moderate dementia), 10–14 (moderately severe dementia) and 0–9 (severe dementia). The MMSE has demonstrated validity and reliability in geriatric, psychiatric, neurological and other medical populations [23], also in the Netherlands [24].

#### EQ-5D and EQ-5D+C

The EQ-5D is a generic instrument to measure HRQoL. The instrument was developed and validated in a number of European countries including the Netherlands [3,25,26]. The EQ-5D describes health status according to five dimensions: mobility, self-care, usual activities, pain/discomfort and anxiety/depression. Each dimension has three levels, namely, "no problems", "some problems" and "severe problems". This yields 243 potential combinations of health states across the five dimensions. Dolan et al. [27] have presented 42 of these health states to members of a representative sample of the UK general population, which were valued using the TTO method. Based on these valuations, utility scores can be deduced by means of an additive function. These are now widely used in cost-utility analyses [28]. Utility scores can vary between -0.59 (worst health) and 1.00 (perfect health). Besides the five dimensions, the EQ-5D consists of a visual analogue scale (VAS_5D_) ranging from 0 (worst imaginable health state) to 100 (best imaginable health state).

The EQ-5D+C is an extended version of the EQ-5D that includes "cognitive functioning" (memory, concentration, coherence, IQ) as an additional dimension, with a similar operationalization of three levels (as described above) [15]. The EQ-5D+C also includes a VAS_5D_.

In this study, the EQ-5D was administered completely first, that is the five dimensions followed by the VAS_5D_. Subsequently, the proxies were asked to answer the sixth dimension concerning cognitive functioning, whereupon the VAS_5D _was valued a second time (in this study referred to as the VAS_5D+C_). Therefore, in this study, the EQ-5D+C refers to the additional cognitive functioning dimension and the VAS_5D+C_.

### Data analysis

#### Construct validity

Construct validity, the extent to which an instrument correlates with other measures which it should be related to [29], was estimated by studying correlations between the EQ-5D and the MMSE and between the EQ-5D+C and the MMSE at baseline and follow-up measures. Although it was expected that effects of cognitive impairment were implicitly expressed in the EQ-5D dimensions concerning self-care, usual activities and anxiety/depression [18,30], we hypothesized that the correlations between the EQ-5D+C and the MMSE were strongest.

#### Responsiveness

In this study, responsiveness was defined as the correlation of the changes in an instrument to changes in other measures which it should be related to, using an anchor-based approach [31,32]. We evaluated whether changes in the EQ-5D and changes in the EQ-5D+C correlated with changes in the MMSE, the so-called anchor (external standard). Again, it was hypothesized that the correlations between the EQ-5D+C and the MMSE were stronger than the correlations between the EQ-5D and the MMSE.

### Statistical analysis

The software used for the analyses was SPSS version 12.0.1 and STATA version 8.2. Background characteristics of the participants (both the patients and their proxies) were summarized using descriptive statistics. Response distributions of the instruments (EQ-5D, EQ-5D+C and MMSE) are given. Missing data of the participants were imputed using multiple imputation (MI). MI provides a useful strategy for dealing with data sets with missing values. Instead of filling in a single value for each missing value, Rubin's [33] multiple imputation procedure replaces each missing value with a set of plausible values (5) that represent the uncertainty about the right value to impute. This results in statistically valid inferences that properly reflect the uncertainty due to missing values.

Kolmogorov-Smirnov tests were used to test for normality. Non-parametric tests for comparisons were selected. Associations between the instruments were analyzed with Spearman rank correlations.

## Results

### Sample characteristics

Table 1 summarizes sample characteristics. Of the 234 patients that were included in the MEDICIE-study, 64.1% were females. Of the proxies, 66.7% were females and mostly children (-in-law) or spouses of the patient (90.2%). In most cases, dementia (present in 70.1% of the patients) was associated with Alzheimer's disease (41.5%). Patients whose etiological diagnoses could not be determined were assigned to the "other" groups (i.e. other dementia or other cognitive impairment).

**Table 1:** Sample characteristics at baseline

	**Total n = 234**
**Gender of patient:**	
Female (%)	150 (64.1%)
**Age of patient:**	
Mean (SD)	77.8 (6.7)
Range	[55–94]
**Relationship proxy:**	
Spouse	88 (37.6%)
Child (-in-law)	123 (52.6%)
Other	23 (9.8%)
**Gender of proxy:**	
Female (%)	156 (66.7%)
**Age of proxy:**	
Mean (SD)	59.8 (13.9)
Range	[30–91]
**MMSE:**	
Mean (SD)	20.18 (5.8)
**Dementia:**	**164 (70.1%)**
Alzheimer's Disease (AD)	97 (41.5%)
Vascular Dementia (VD)	26 (11.1%)
Mixed Dementia	21 (9.0%)
Other Dementia	20 (8.5%)
**No Dementia:**	**70 (29.9%)**
Cognitive Impairment/MCI	40 (17.1%)
Other Cognitive Impairment	30 (12.8%)

After six months, 16 patients (6.8%) had died and 11 patients (4.7%) and their caregivers had dropped out of the study. Four patients (1.7%) did not attend the six month follow-up because of personal reasons. After 12 months another 11 patients (4.7%) had died and two more patients (0.9%) and their caregivers had dropped out of the study. The 27 patients (11.5%) who had died were excluded from the analyses as well as the 11 study dropouts (4.7%) who completed only 1 measurement. Missing data of the remaining 196 patients were imputed using MI. To make sure the imputations did not influence our results, separate analyses were performed on the 5 imputed datasets. The results were highly comparable (data not shown). Therefore, the first imputed dataset was used for analysis.

### Responses in the EQ-5D, EQ-5D+C and MMSE

The responses in the EQ-5D and the EQ-5D+C at baseline and follow-up measurements are summarized in table 2. At baseline as well as at the 6 month follow-up, most patients had problems with cognitive functioning, usual activities and mobility. At the 12 month follow-up, most patients had problems with cognitive functioning, usual activities and self-care. The mean VAS_5D+C _scores were significantly lower than the mean VAS_5D _scores at all measurements (Wilcoxon Signed Ranks Tests, p = 0.000).

**Table 2:** Responses in the EQ-5D and EQ-5D+C (items, utility scores and VAS scores)

**EQ-5D N = 196**	**Baseline**	**6 months**	**12 months**
**Mobility:**			
Mean (SD)	1.65 (0.48)	1.65 (0.51)	1.67 (0.51)
% no problems	24.7%	36.7%	34.7%
% some problems	65.3%	61.7%	63.3%
% severe problems	0%	1.5%	2.0%
**Self-Care:**			
Mean (SD)	1.59 (0.65)	1.71 (0.73)	1.97 (0.80)
% no problems	50.0%	45.4%	33.2%
% some problems	40.8%	38.3%	36.7%
% severe problems	9.2%	16.3%	30.1%
**Usual Activities:**			
Mean (SD)	1.92 (0.72)	2.02 (0.75)	2.17 (0.78)
% no problems	29.6%	27.0%	23.0%
% some problems	48.5%	44.4%	36.7%
% severe problems	21.9%	28.6%	40.3%
Pain/Discomfort:			
Mean (SD)	1.59 (0.65)	1.51 (0.60)	1.54 (0.62)
% no problems	49.5%	54.1%	53.1%
% some problems	41.8%	40.8%	40.3%
% severe problems	8.7%	5.1%	6.6%
**Anxiety/Depression:**			
Mean (SD)	1.66 (0.67)	1.53 (0.59)	1.54 (0.68)
% no problems	44.9%	52.6%	56.6%
% some problems	43.9%	42.3%	33.2%
% severe problems	11.2%	5.1%	10.2%
			
**Utility score:**			
Mean (SD)	0.54 (0.31)	0.57 (0.32)	0.47 (0.34)
Range	-0.35 – 1.00	-0.35 – 1.00	-0.43 – 1.00
**VAS**_5D_**:**			
Mean (SD)	58.84 (19.00)	57.78 (17.67)	56.38 (20.29)
Range	10–100	20–100	10–100

**EQ-5D+C**			

**Cognition:**			
Mean (SD)	2.30 (0.58)	2.35 (0.64)	2.47 (0.61)
% no problems	6.1%	8.7%	6.1%
% some problems	58.2%	47.4%	40.8%
% severe problems	35.7%	43.9%	53.1%
			
**VAS**_5D+C_**:**			
Mean (SD)	49.45 (19.23)	48.43 (18.06)	45.41 (18.90)
Range	0 – 100	10 – 90	0 – 95

The responses in the MMSE at baseline and follow-up measurements are summarized in table 3. Most patients had mild to moderate dementia at all measurements.

**Table 3:** Responses in the MMSE

**N = 196**	**Baseline**	**6 months**	**12 months**
**MMSE**			
Mean (SD)	20.21 (5.78)	18.89 (7.34)	17.61 (8.16)
**Severity of dementia:**			
26 – 30 (normal ageing)	18.4%	21.9%	21.9%
21 – 25 (mild)	35.7%	26.0%	19.4%
15 – 20 (moderate)	29.6%	25.0%	25.0%
10 – 14 (moderately severe)	10.2%	14.3%	15.3%
0 – 9 (severe)	6.1%	12.8%	18.4%

### Construct validity

Table 4 summarizes the results of the correlations between the MMSE and the EQ-5D and between the MMSE and the EQ-5D+C. At baseline, significant correlations were found between the MMSE and the utility score, and more specifically the self-care dimension and the usual activities dimension, and the VAS_5D _of the EQ-5D. Correlations were also found between the MMSE and the cognitive dimension and the VAS_5D+C _of the EQ-5D+C. At the six month follow-up, correlations were found between the MMSE and the utility score, and more specifically all five dimensions, and the VAS_5D _of the EQ-5D. Correlations were also found between the MMSE and the cognitive dimension and the VAS_5D+C _of the EQ-5D+C. At the 12 month follow-up, correlations were found between the MMSE and the utility score, and more specifically all dimensions except for the pain/discomfort dimension, and the VAS_5D _of the EQ-5D. Correlations were also found between the MMSE and the cognitive dimension and the VAS_5D+C _of the EQ-5D+C.

**Table 4:** Spearman correlations between the EQ-5D and the MMSE and between the EQ-5D+C and the MMSE at baseline, six months and 12 months (construct validity)

**N = 196**	**Baseline**	**6 months**	**12 months**
**EQ-5D:**			
Utility score	0.19**	0.45**	0.50**
Mobility	-0.02	-0.17*	-0.23**
Self-Care	-0.28**	-0.50**	-0.55**
Usual activities	-0.34**	-0.42**	-0.52**
Pain/Discomfort	-0.01	-0.14*	-0.05
Anxiety/Depression	-0.05	-0.17*	-0.22**
			
VAS_5D_	0.22**	0.23**	0.23**
			
**EQ-5D+C:**			
			
Cognition	-0.35**	-0.52**	-0.54**
VAS_5D+C_	0.37**	0.47**	0.48**

** Correlation is significant at the 0.01 level (2-tailed)

* Correlation is significant at the 0.05 level (2-tailed)

### Responsiveness

Table 5 summarizes the results of the correlations between the change scores of the EQ-5D and the EQ-5D+C and the change scores of the MMSE. In table 6, the means for the change scores are outlined. Regarding the difference between the six month measurement and the baseline measurement, correlations were found between changes in the utility score, more specifically the self-care dimension, of the EQ-5D and the change scores of the MMSE. Correlations were also found between changes in the cognitive dimension of the EQ-5D+C and change scores of the MMSE. Regarding the difference between the 12 month measurement and the baseline measurement, correlations were found between changes in the utility score, more specifically the mobility dimension, the self-care dimension and the usual activities dimension, and the VAS_5D _of the EQ-5D and the change scores of the MMSE. Correlations were also found between changes in the cognitive dimension and the VAS_5D+C _of the EQ-5D+C and the change scores of the MMSE.

**Table 5:** Spearman correlations between the change scores of the EQ-5D and the MMSE, and between the EQ-5D+C and the MMSE, i.e. change score of baseline and six months and baseline and 12 months (longitudinal)

**Change scores N = 196**	**Baseline & 6 months**	**Baseline & 12 months**
**EQ-5D:**		
Utility score	0.16*	0.30**
Mobility	-0.11	-0.17*
Self-Care	-0.18*	-0.35**
Usual activities	-0.12	-0.29**
Pain/Discomfort	-0.04	0.03
Anxiety/Depression	0.00	-0.12
		
VAS_5D_	0.01	0.17*
		
**EQ-5D+C:**		
Cognition	-0.21**	-0.28**
VAS_5D+C_	0.08	0.23**

** Correlation is significant at the 0.01 level (2-tailed)

* Correlation is significant at the 0.05 level (2-tailed)

**Table 6:** Means of the change scores of the EQ-5D, the EQ-5D+C and the MMSE

**Change scores N = 196**	**Baseline & 6 months Mean (SD)**	**Baseline & 12 months Mean (SD)**
**EQ-5D:**		
Utility score	0.02 (0.29)	-0.07 (0.31)
		
Mobility	-0.01 (0.44)	0.02 (0.53)
Self-Care	0.12 (0.58)	0.38 (0.71)
Usual activities	0.09 (0.70)	0.25 (0.69)
Pain/Discomfort	-0.08 (0.58)	-0.06 (0.67)
Anxiety/Depression	-0.14 (0.66)	-0.13 (0.74)
		
VAS_5D_	-1.06 (18.00)	-2.46 (21.40)
		
**EQ-5D+C:**		
Cognition	0.06 (0.63)	0.17 (0.66)
VAS_5D+C_	-1.02 (18.36)	-4.05 (21.33)
		
**MMSE:**		
Mean (SD)	-1.32 (4.58)	-2.61 (5.17)

## Discussion

The aim of this explorative study was to compare the performance of the EQ-5D and the EQ-5D+C by assessing their construct validity and responsiveness in patients aged 55 and older with cognitive impairments.

Based on our results it can be concluded that the construct validity of the EQ-5D and the EQ-5D+C is comparable in our study population, except for the VAS_5D_. Results regarding construct validity of the EQ-5D are in line with the recent findings of Jönssen et al. [17]. Contrary to our expectations, correlations between the cognitive dimension and the MMSE were almost similar to the correlations between the self-care and the usual activities dimensions and the MMSE. The presence of more and stronger correlations of both the EQ-5D and EQ-5D+C with the MMSE at the 12 month follow up measurement was possibly due to the fact that the dispersion of the scores using these instruments increased with time. Three studies also showed that cognitive function was positively related to HRQoL in cardiac rehabilitation patients [34], in patients with progressive supranuclear palsy [35] and in patients with hypertension [36]. Another study [37] failed to find a relationship between HRQoL and cognition in patients with dementia.

With regard to responsiveness, the EQ-5D performed slightly better than the EQ-5D+C, which is also in line with the findings of Jönssen [17]. An important finding, again contrary to our expectations, is that changes in the MMSE corresponded better with changes in the self-care dimension and the usual activities dimension than with changes in the cognitive dimension.

However, no judgments were made about the strength of the correlations, which would provide us with a stricter criterion regarding the performance of the EQ-5D and EQ-5D+C. In the literature, different classifications were found [38-41] a clear gold standard being absent. We therefore ignored the classifications and merely described our results. However, it is possible to compare our results with other studies. Our results were in line with correlations between the EQ-5D and clinical measures found in other studies involving diseases such as progressive supranuclear palsy (PSP) [35], rheumatoid arthritis (RA) [39] and stroke [42].

The majority of authors [39,41,42] and others) considered a Spearman's correlation of > 0.50 to be strong, a correlation of 0.30/0.35 – 0.50 to be moderate and a correlation < 0.30/0.35 to be weak. Using these classifications in our study, it can be concluded that both versions performed well with respect to construct validity, as indicated by strong correlations with the MMSE. Regarding responsiveness, it can be concluded that the EQ-5D performed moderately, whereas the EQ-5D+C did less well as indicated by weak correlations with the MMSE. When the more stringent classification of Landis and Koch [38] is used (i.e. < 0.00 poor; 0.00–0.20 slight; 0.21–0.40 fair; 0.41–0.60 moderate; 0.61–0.80 substantial and 0.81–1.00 almost perfect), it can be concluded that the EQ-5D and the EQ-5D+C performed moderately with regard to the construct validity.

Regarding responsiveness, fair correlations were found between changes in the EQ-5D and EQ-5D+C and changes in the MMSE. The relatively low responsiveness of the EQ-5D in this study could be due to the, on average, small changes in cognition in a year, or to a ceiling effect because there are only three levels for each dimension of the EQ-5D. Patients' health may improve or decline but not enough to go up or down one level. Instruments that have a greater number of possible responses may be more responsive. Furthermore, it is possible that adaptation to illness on the part of the proxy leads to a lack of responsiveness, especially with a chronic condition such as dementia [39]. It should also be noted that a lack of clarity exists with regard to the definition and adequate approach for evaluating responsiveness. Some authors argued that there is no need for an additional concept like responsiveness, since it can be viewed as either longitudinal validity or magnitude of the treatment effect [32,43,44]. The definition and approach used in this study has also been referred to as longitudinal validity [32].

There are several limitations to this study that need to be recognized. An important limitation of this study concerns our study design. The origin of this study, the MEDICIE trial, was designed to compare the effects of a multidisciplinary diagnostic observation centre for psychogeriatric patients (DOC-PG) with care as usual on HRQoL, mental and physical health, and the costs and use of health care facilities by patients with psychogeriatric problems. Therefore, studying the usefulness of the EQ-5D+C in this patient population was framed in this RCT. The EQ-5D was administered first, that is the five dimensions followed by the VAS_5D_. Subsequently, the proxies were asked to answer the sixth dimension concerning cognitive functioning, whereupon the VAS_5D+C _was valued. It would have been better to administer the EQ-5D+C completely as well in order to make valid comparisons between the 2 versions. However, considering the explorative nature of this study, we did not want to burden the participants of the MEDICIE trial by administering a similar questionnaire twice.

Second, regarding the assessment of the EQ-5D+C, the proxies may have focused their attention on the cognitive dimension when scoring the VAS_5D+C_, even though they had been instructed to rate the VAS_5D+C _again based on the overall health. This effect is called a framing effect, which suggests that how something is presented (the 'frame') influences the choices people make [45]. Hence, it is possible that the higher correlations of the VAS_5D+C _with the MMSE are due to a framing effect. However, according to Parkin et al. [46], the framing bias also exists when assessing the EQ-5D, meaning that values of the VAS_5D _are affected by end-state descriptors (last named dimensions).

Another possible limitation is the use of proxies to complete the questionnaires. Previous research indicated that there is generally fairly good proxy-patient agreement for observable items such as mobility, self care and usual activities, but poor agreement for non-observable items such as pain and affect [16]. Others have found agreement to be poor for the domains most affected by dementia (self-care and usual activities) [17]. In the light of the longitudinal nature of our study, the complex health problems of our study population and their progressive global deterioration of intellect and personality, the method of proxy rating had been chosen. It is generally acknowledged that in the later stages of dementia proxy measures are required since patients are no longer capable of making an adequate evaluation of their HRQoL [12,17]. Furthermore, the use of proxy reports throughout the course of a longitudinal study, rather than substituting them only when the person with dementia becomes unable to report his or her HRQoL, reduces bias over time [47]. The overall picture of previous research is that rating by proxy is a valid alternative for assessing HRQoL in the presence of dementia [17,47-49], although it is possible that the scores in the EQ-5D and the EQ-5D+C were biased because of perceived caregiver burden [50].

A final limitation also concerns the design of our study. Comparisons between the EQ-5D and the EQ-5D+C were merely based on the dimensions and VAS-scores of both versions and not on the utility scores since these are not available for the EQ-5D+C. It should be noted that an algorithm has been developed for EQ-5D+C health states, based on Dutch disability weights [51,52]. In the Dutch disability weights study, a comprehensive set of disease-specific disability weights for 175 disease stages associated with 52 disease categories was obtained [53,54]. Based on these disability weights, an EQ-5D+C regression model was fitted. However, the origins of the EQ-5D+C disability weights and the EQ-5D utility scores differ significantly. First, the algorithm is based on valuations of health experts instead of valuations of the general public. Second, EQ-5D+C health states were valued by means of the person trade-off (PTO) method, whereas EQ-5D health states were valued by means of the time trade-off (TTO) method [27]. PTO differs from TTO in that subjects are required to trade-off person years lived healthy against person years lived with some defined disability, thus making choices in the context of a decision involving other people rather than themselves. Whether the PTO technique is able to reflect actual preferences is still under debate [55,56]. Finally, besides the EQ-5D+C health state description, subjects were given specific information with respect to the disease, which differs from the EQ-5D valuation procedure [27]. Therefore, in our opinion, no valid comparison of EQ-5D+C disability weights with EQ-5D utility scores can be made. In order to develop a new scoring algorithm of which utility scores for the EQ-5D+C can be deduced, a valuation procedure similar to the one used for the EQ-5D should be applied. Presenting EQ-5D+C health states to members of the general population should reduce the framing effect described earlier, as the cognitive dimension will then be 'just' one of the six dimensions in the health states. Furthermore, although in the descriptive part of the EQ-5D a proxy effect may still be present, by using a utility score based on valuations of the general population, possible proxy effects are expected to decrease.

## Conclusion

In this explorative study, the construct validity and responsiveness of the EQ-5D and the EQ-5D+C were assessed and compared in patients aged 55 and older with cognitive impairments. We conclude that the EQ-5D performs well for evaluating HRQoL in our population with cognitive impairments using proxy ratings. Therefore, based on the results of this study and given its (serious) limitations, it does not seem necessary to adjust the current classification system by adding a cognitive dimension. However, in the absence of a gold standard for measuring HRQoL, a general population study to obtain valuations for the EQ-5D+C health states could provide a better insight into whether cognition has a separate and significant effect on utility values, and would enable us to compare the utility values deduced from both versions in a correct manner.

## Competing interests

The author(s) declare that they have no competing interests.

## Authors' contributions

FV was the principal investigator of the larger trial and is guarantor. FV, AK, CD, and JS designed this trial. CW and DW carried out the outcome measures. AK and CW were responsible for statistical analysis and interpretation of the study, and all authors contributed to interpretation. CW drafted the manuscript, and all authors critically revised it for scientific content and approved the final version.
